# Supplementary material for: Lung Epithelial CYP1 Activity Regulates Aryl Hydrocarbon Receptor Dependent Allergic Airway Inflammation
Source: Front Immunol. 2022 Jun 6;13:901194. doi: 10.3389/fimmu.2022.901194 (PMC9207268; doi:10.3389/fimmu.2022.901194)
Supplement: Supplementary file 1 [file Presentation_1.pptx]

## Slide 1
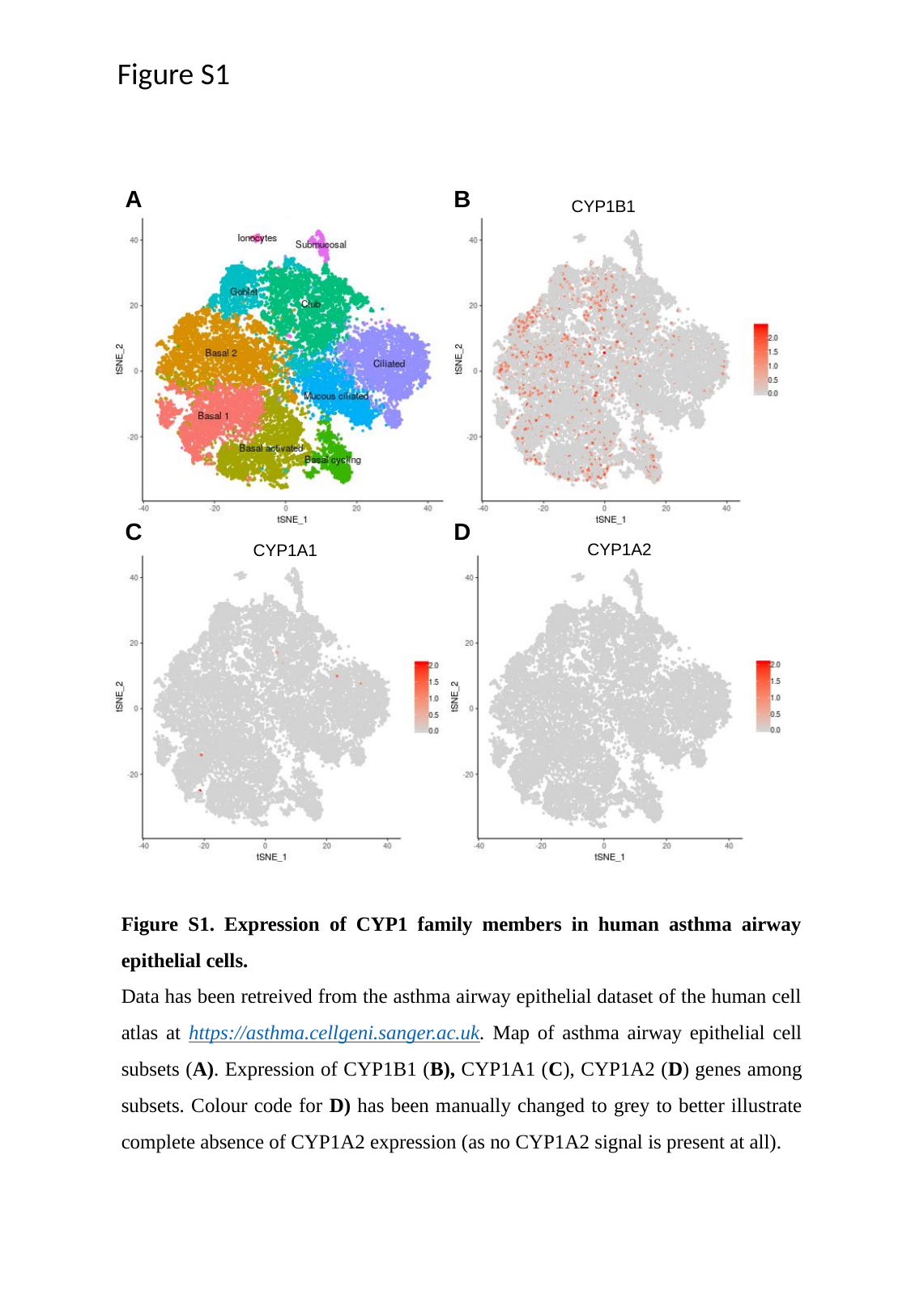

Figure S1
A
B
CYP1B1
C
D
CYP1A2
CYP1A1
Figure S1. Expression of CYP1 family members in human asthma airway epithelial cells.
Data has been retreived from the asthma airway epithelial dataset of the human cell atlas at https://asthma.cellgeni.sanger.ac.uk. Map of asthma airway epithelial cell subsets (A). Expression of CYP1B1 (B), CYP1A1 (C), CYP1A2 (D) genes among subsets. Colour code for D) has been manually changed to grey to better illustrate complete absence of CYP1A2 expression (as no CYP1A2 signal is present at all).

## Slide 2
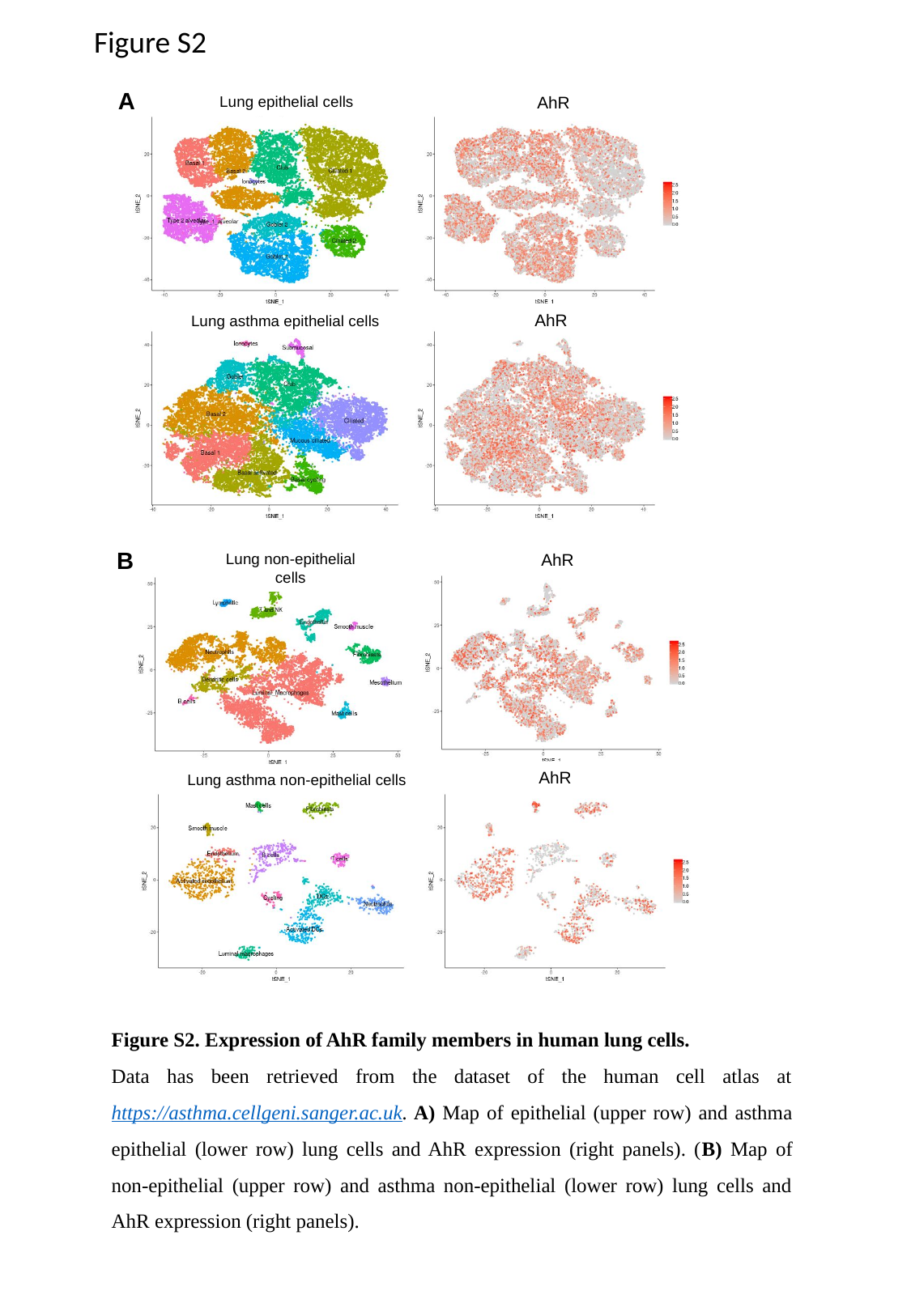

Figure S2
A
Lung epithelial cells
AhR
AhR
Lung asthma epithelial cells
B
Lung non-epithelial cells
AhR
AhR
Lung asthma non-epithelial cells
Figure S2. Expression of AhR family members in human lung cells.
Data has been retrieved from the dataset of the human cell atlas at https://asthma.cellgeni.sanger.ac.uk. A) Map of epithelial (upper row) and asthma epithelial (lower row) lung cells and AhR expression (right panels). (B) Map of non-epithelial (upper row) and asthma non-epithelial (lower row) lung cells and AhR expression (right panels).

## Slide 3
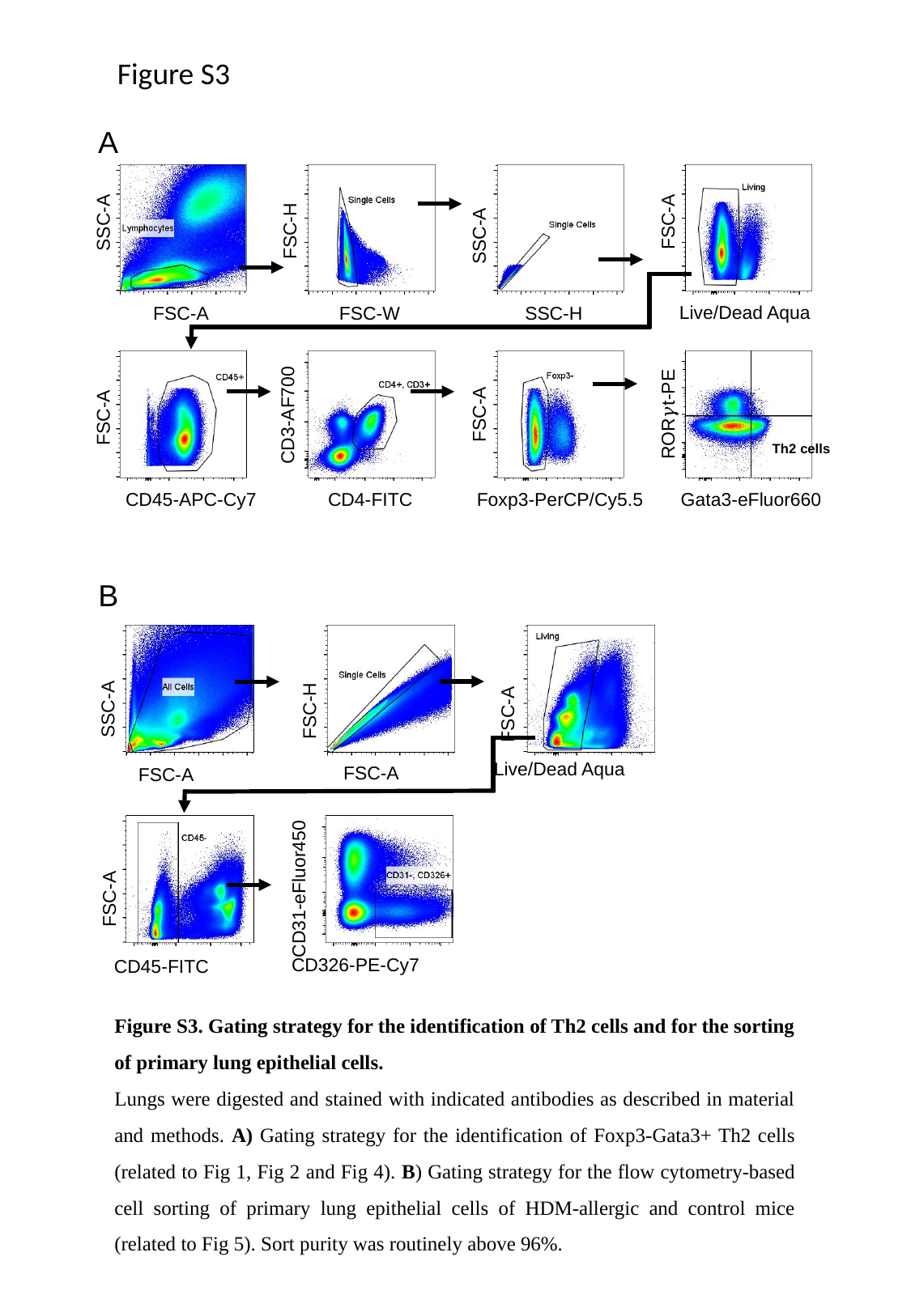

Figure S3
A
FSC-A
SSC-A
FSC-H
SSC-A
Live/Dead Aqua
FSC-A
FSC-W
SSC-H
FSC-A
ROR𝛾t-PE
CD3-AF700
FSC-A
Th2 cells
CD45-APC-Cy7
CD4-FITC
Foxp3-PerCP/Cy5.5
Gata3-eFluor660
B
SSC-A
FSC-H
FSC-A
Live/Dead Aqua
FSC-A
FSC-A
CD31-eFluor450
FSC-A
CD326-PE-Cy7
CD45-FITC
Figure S3. Gating strategy for the identification of Th2 cells and for the sorting of primary lung epithelial cells.
Lungs were digested and stained with indicated antibodies as described in material and methods. A) Gating strategy for the identification of Foxp3-Gata3+ Th2 cells (related to Fig 1, Fig 2 and Fig 4). B) Gating strategy for the flow cytometry-based cell sorting of primary lung epithelial cells of HDM-allergic and control mice (related to Fig 5). Sort purity was routinely above 96%.
