# Supplementary material for: Lung Epithelial CYP1 Activity Regulates Aryl Hydrocarbon Receptor Dependent Allergic Airway Inflammation
Source: Front Immunol. 2022 Jun 6;13:901194. doi: 10.3389/fimmu.2022.901194 (PMC9207268; doi:10.3389/fimmu.2022.901194)
Supplement: Supplementary file 2 [file Table_1.docx]

| **Marker** | **Fluorophore** | **Clone** | **Source** | **Catalogue number** |
| --- | --- | --- | --- | --- |
| Fc Block | - | 2.4G2 | BD Biosciences | #553141 |
| CD45 | FITC | 30-F11 | eBiosciences | #11-0451-82 |
| CD45 | APC-eFluor780 | 30-F11 | eBiosciences | #47-0451-82 |
| CD31 | eFluor450 | 390 | eBiosciences | #48-0311-82 |
| CD326 (EpCam) | PE-Cy7 | G8.8 | eBiosciences | #25-5791-80 |
| CD3 | FITC | 145-2C11 | BD Biosciences | #553062 |
| CD3 | AlexaFluor700 | 17A2 | BioLegend | #100216 |
| CD4 | FITC | GK1.5 | BD Biosciences | #553729 |
| CD4 | AlexaFluor700 | RM4-5 | BD Biosciences | #557956 |
| Foxp3 | PerCP/Cy5.5 | FJK-16s | BD Biosciences | #45-5773-82 |
| Gata3 | eFluor660 | TWAJ | eBiosciences | #50-9966-42 |
| RORγt | PE | AFKJS-1 | eBiosciences | #12-6988-82 |
| Live/Dead | Zombie Aqua | - | BioLegend | #423101 |

**Table S1.** Antibodies used for flow cytometry
