# Supplementary material for: Lung Epithelial CYP1 Activity Regulates Aryl Hydrocarbon Receptor Dependent Allergic Airway Inflammation
Source: Front Immunol. 2022 Jun 6;13:901194. doi: 10.3389/fimmu.2022.901194 (PMC9207268; doi:10.3389/fimmu.2022.901194)
Supplement: Supplementary file 3 [file Table_2.docx]

**Table S2.** Primers used for real-time PCR

| IL-4 | Forward:  Reverse: | GTC ATC CTG CTC TTC TTT CTC G  CTC TCT GTG GTG TTC TTC GTT G |
| --- | --- | --- |
| IL-13 | Forward:  Reverse: | CCT GGC TCT TGC TTG CCT T  GGT CTT GTG TGA TGT TGCT CA |
| IFN-γ | Forward:  Reverse: | GCA TTC ATG AGT ATT GCC AAG  GGT GGA CCA CTC GGA TGA |
| IL-17a | Forward:  Reverse: | TCC AGA AGG CCC TCA GAC TA  TGA GCT TCC CAG ATC ACA GA |
| AhR | Forward:  Reverse: | TCT GTT CTT AGG CTC AGC GTC  GCG CCT GTA ACA AGA ACT CTC |
| CYP1A1 (Qiagen) | Forward:  Reverse: | Mm_Cyp1a1_1_SG QuantiTect Primer Assay |
| CYP1B1 (Qiagen) | Forward:  Reverse: | Mm_Cyp1b1_2_SG QuantiTect Primer Assay |
| CCL11 | Forward:  Reverse: | GGC TGA CCT CAA ACT CAC AGA AA  ACA TTC TGG CTT GGC ATG GT |
| GAPDH | Forward:  Reverse | CGT CCC GTA GAC AAA ATG GT  TTG ATG GCA ACA ATC TCC AC |
| Beta-actin | Forward:  Reverse | TTC TTT GCA GCT CCT TCG TT  ATG GAG GGG AAT ACA GCC C |
